# Air Pollution and Birth Weight: Bell et al. Respond

**DOI:** 10.1289/ehp.10631R

**Published:** 2008-03

**Authors:** Michelle L. Bell, Keita Ebisu, Kathleen Belanger

**Affiliations:** School of Forestry and Environmental Studies, Yale University, New Haven, Connecticut, E-mail: michelle.bell@yale.edu; Department of Epidemiology and Public Health, School of Medicine, Yale University, New Haven, Connecticut

In our recent study (Bell et al. 2007) we identified associations between air pollution and low birth weight in Connecticut and Massachusetts based on 358,504 births from 1999 to 2002. Salam raises several important concerns about potential limitations in the analysis and interpretation of our results. In particular, he discusses gestational period, effects by race, confounding by co-pollutants, and competing biological mechanisms among other issues.

We conducted several sensitivity analyses as suggested by Salam in his letter, and detailed results are available upon request. The original study considered confounding by co-pollutants for pairs of pollutants that were not highly correlated, and found that model results were robust to adjustment by other pollutants. Salam correctly notes that pollutant data were not available from all counties; therefore, a given observation may have data for some exposure variables and not others. We performed a new analysis comparing results based only on observations with data for the two pollutants considered for gestational exposure. For example, we calculated the association between particulate matter < 2.5 μm in aerodynamic diameter (PM_2.5_) and birth weight, adjusted by carbon monoxide, and the association between PM_2.5_ and birth weight not adjusted by CO, but including only the subset of observations with CO data available. The new analysis did not change the results or interpretation.

In our initial analysis we omitted births with gestational length < 32 weeks or > 44 weeks, and adjusted for gestational length at 2-week intervals. As noted in our article (Bell et al. 2007), births with gestational length of 32–36 weeks accounted for 6.7% of the original observations. Salam proposed analysis of gestational and third-trimester exposure restricted to observations with 37–44 weeks gestation. We performed this analysis and generated effect estimates for first- and second-trimester exposure as well. Effects estimates based on the subset analysis (37–44 weeks) were very similar to those from the original analysis.

Salam notes that combining non-Hispanic and Hispanic whites, as done in our study, does not allow for distinction between Hispanics and non-Hispanics, and that race may be associated with socioeconomic status. Although we controlled for race and for socioeconomic status (through mother’s education), we agree that the analysis has limitations. Research of effects by race is further complicated by distinction of non-Hispanic black versus Hispanic black and by other subdivisions of racial and ethnic categories (e.g., Mexican vs. Cuban heritage), as well as multiracial infants. Other restrictions may arise from lack of sufficient sample size to investigate various racial categories. To date, few low birth weight and air pollution studies have specifically investigated race (e.g., Basu et al. 2004; Bell et al. 2007; Maisonet et al. 2001), although others have included race as a covariate or restricted observations by race.

Although Salam’s letter is directed at an individual study, it highlights some of the challenges of air pollution and pregnancy outcome studies more broadly. Many epidemiologic studies evaluate exposure from monitoring networks implemented for regulatory compliance, and not all areas have monitors. Multipollutant analysis is additionally complex because of the high correlation of some pollutants and by the variation in the chemical composition of particulate matter. Factor analysis and other source apportionment techniques (e.g., Thurston et al. 2005) that have been used to investigate the association between particles and other health outcomes may be used to estimate exposure based on sources for birth outcomes research. A recent study of 1,016 births in the Munich, Germany, metropolitan area assessed exposure to traffic-related pollution accounting for PM_2.5_ and nitrogen dioxide levels, land use, road characteristics, and population density (Slama et al. 2007).

A critical question is the biological mechanism through which air pollution affects fetal growth, and the potential competing mechanisms of impacts on preterm delivery and fetal growth, as mentioned by Salam. He also notes that other useful results would include analyses of mothers who smoke and of counties with a higher proportion of people living in poverty. In our study (Bell et al. 2007), we adjusted for mother’s smoking and education, as indicators of socioeconomic status, in all models; however, we did not investigate the effect modification of smoking or economic conditions. Although such a study would be informative, our data set is not well suited to this analysis because of the lack of extensive data on smoking or socioeconomic status. More detailed and accurate information on these variables may be available from cohort studies.

Limitations of the current scientific literature on birth outcomes and air pollution prompted two recent international workshops, the International Workshop on Air Pollution and Human Reproduction in Munich in May 2007, and the Methodological Issues in Studies of Air Pollution and Perinatal Outcomes Workshop in Mexico City in September 2007. These workshops explored exposure assessment, confounding and effect modification, the relevant window of exposure before or during pregnancy, biological mechanisms, spatial analysis, and the public health implications of observed associations. Reports from the workshops are forthcoming.
